# microRNA-4717 differentially interacts with its polymorphic target in the *PD1* 3′ untranslated region: A mechanism for regulating PD-1 expression and function in HBV-associated liver diseases

**DOI:** 10.18632/oncotarget.3662

**Published:** 2015-03-26

**Authors:** Guoyu Zhang, Na Li, Zhu Li, Qianqian Zhu, Fang Li, Cuiling Yang, Qunying Han, Yi Lv, Zhihua Zhou, Zhengwen Liu

**Affiliations:** ^1^ Department of Infectious Diseases, First Affiliated Hospital, School of Medicine, Xi'an Jiaotong University, Xi' an, Shaanxi, China; ^2^ Department of Hepatobiliary Surgery, First Affiliated Hospital, School of Medicine, Xi'an Jiaotong University, Xi'an, Shaanxi, China; ^3^ Institute of Advanced Surgical Technology and Engineering, Xi'an Jiaotong University, Xi' an, Shaanxi, China

**Keywords:** programmed cell death-1, 3′-untranslated region, single nucleotide polymorphism, microRNA, hepatitis B virus infection

## Abstract

Programmed cell death-1 (PD-1) is involved in hepatitis B virus (HBV) infection, the leading cause of hepatocellular carcinoma (HCC) worldwide. Single-nucleotide polymorphism, rs10204525, located in the *PD1* 3′ untranslated regions (UTR), is associated with chronic HBV infection. MicroRNAs (miRNAs) regulate gene expression via specific binding to the target 3′UTR of mRNA. In this study, three miRNAs were predicted to putatively interact with *PD1* rs10204525 polymorphic site of allele G. One of them, miRNA-4717, was demonstrated to allele-specifically affect luciferase activity in a dose-dependent manner in cells transfected with vectors containing different rs10204525 alleles. In lymphocytes from chronic HBV patients withrs10204525 genotype GG, miR-4717 mimics significantly decreased PD-1 expression and increased (TNF)-α and interferon (IFN)-γ production. miR-4717 inhibitor significantly increased PD-1 expression and decreased TNF-α and IFN-γ production although not significantly. In lymphocytes from chronic HBV patients with rs10204525 genotype AA, no similar effects were observed. miR-4717 levels in peripheral lymphocytes from patients with HBV-related chronic hepatitis, cirrhosis and HCC were significantly decreased. In conclusion, miR-4717 may allele-specifically regulate PD-1 expression through interaction with the 3′ UTR of *PD1* mRNA, leading to the alteration of immune regulation and affecting the susceptibility and disease course of chronic HBV infection.

## INTRODUCTION

Infection with hepatitis B virus (HBV) remains a major public health problem. It is estimated that more than 350 million people are HBV carriers worldwide and about one million annual deaths are resulted from HBV-related liver diseases such as cirrhosis and hepatocellular carcinoma (HCC) [1]. The liver damage and disease progression of HBV infection is primarily associated with immunoinflammatory responses of the host to the virus [2-5].

Programmed cell death-1 (PD-1) is a prominent regulator of T cell function and a major factor of apoptosis sensitivity [6, 7]. Engagement of PD-1 by its ligands PD-L1 or PD-L2 could inhibit T-cell receptor-mediated proliferation and cytokine production in activated T lymphocytes [8]. The pathway of PD-1 and its ligands has been increasingly demonstrated to be substantially involved in viral infection, including HBV infection. Specifically, viral infection and interferon (IFN)-α and IFN-γ could induce PD-L1 in hepatocytes which could mediate T cell apoptosis [9]. Over-expression of PD-1, PD-L1 and PD-L2 in liver was proposed to participate in local immune dysfunction associated to the chronicity of HBV infection and chronic inflammation in chronic HBV infection [10, 11]. Complex T cell immune responses are involved in HBV infection. Studies by HBV transfected mice showed that CD4+ T cells serve as master regulators of the adaptive immune response to HBV, while CD8+ T cells are the key cellular effectors mediating HBV clearance [12]. PD-1 expression in circulating CD4+ and CD8+ T cells is significantly increased in patients with chronic HBV infection [13, 14]. HBcAg was shown to be able to induce PD-1 upregulation on CD4+ T cells in chronic HBV-infected patients [15]. Strong PD-1 upregulation was shown to be basically linked to CD4+ T-cell dysfunction during chronic HBV infection [16]. PD-1 upregulation on peripheral and intrahepatic HBV-specific CD8+ T cells was also shown to be related to the functional suppression of virus-specific CD8+ T lymphocytes in HBV infection [4, 17-20]. In contrast, PD-1 blockade has been revealed to be able to reverse immune dysfunction and viral persistence of HBV infection and increase HBcAg-specific IFN-γ production in intrahepatic T lymphocytes in a mouse animal model [21] and in human HBV infection [20], to partly enhance CD4+ T-cell expansion and IFN-γ and tumor necrosis factor (TNF)-α secretion [16], and to dominantly restore HBV-specific CD8+ T-cell function [22].

PD-1 expression has also been shown to be associated with response to antiviral treatment in chronic HBV infection with its downregulation being associated with favourable treatment outcome [23-25]. Furthermore, in patients with HBV-related HCC, upregulation of circulating PD-1/PD-L1 is associated with poor post-cryoablation prognosis [26] and PD-1+ tumour-infiltrating lymphocytes is correlated with portal vein thrombosis and may serve as a potential prognostic marker [27].

Genetically, single-nucleotide polymorphisms (SNPs), especially SNP rs10204525 (+8669 G/A), in *PD1* gene, have been shown to be associated with the chronicity and disease progression [28], the alteration of cytokine production [29] and the expression of PD-1 in peripheral blood nuclear cells [30] in chronic HBV infection. However, the mechanism by which this *PD1* polymorphism affects the disease course of HBV infection remains largely unclear.

MicroRNAs (miRNAs) are endogenous short noncoding RNAs usually consisting of 20-25 nucleotides. They are functionally involved in multiple biological processes, including development, proliferation, differentiation, apoptosis, and immune responses [31-33]. miRNAs regulate gene expression at the post-transcriptional or translational level by sequence-specific interaction with the 3′-untranslated region (3′ UTR) of targeted mRNA [32, 34]. The SNP rs10204525, which was revealed to be associated with chronic HBV infection [28-30], is located in the 3′ UTR of *PD1* gene. However, whether there are miRNAs that may affect *PD1* gene expression through interaction with the 3′ UTR of *PD1* mRNA in HBV infection is unknown. In the present study, therefore, we predicted miRNAs that may bind to the 3′ UTR of *PD1* mRNA in different genotypes of rs10204525 polymorphism based on bioinformatics and then examined their effect on PD-1 expression using luciferase reporter activity assay. We further investigated the effect of the identified miRNA on PD-1 expression and TNF-α and IFN-γ production in lymphocytes from chronic HBV patients with different rs10204525 genotypes. Lastly, we examined the association of the identified miRNA with the clinical diseases in patients with chronic HBV infection.

## RESULTS

### Predicted miRNAs putatively binding the 3′ UTR of PD1 mRNA

**Figure 1:**
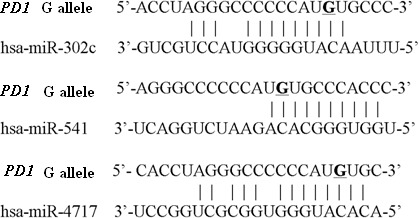
Predicted miRNAs putatively bining the 3′UTR of PD1 mRNA with rs10204525 allele G The *PD1* rs10204525 site is represented by an underscore.

Three miRNAs, namely hsa-miR-302c, hsa-miR-541 and hsa-miR-4717, were predicted to putatively bind the rs10204525 polymorphic site in the 3′ UTR of *PD1* mRNA with allele G (Figure 1) and no miRNA was predicted to putatively bind the 3′ UTR of *PD1* mRNA with allele A by bioinformatics.

### pMIR-REPORT vector identification

Double enzyme digestion with Hind III and Spe I [TAKARA Biotechnology (Dalian) Co., Ltd. Dalian, China] and sequencing confirmed the correctness of the constructs, pMIR*-*A (containing *PD1* rs10204525 A allele) and pMIR*-*G (containing *PD1* rs10204525 G allele) (Supplementary Figure 1, Supplementary Figure 2).

### Effect of miRNAs on PD-1 expression by luciferase activity assay

**Figure 2:**
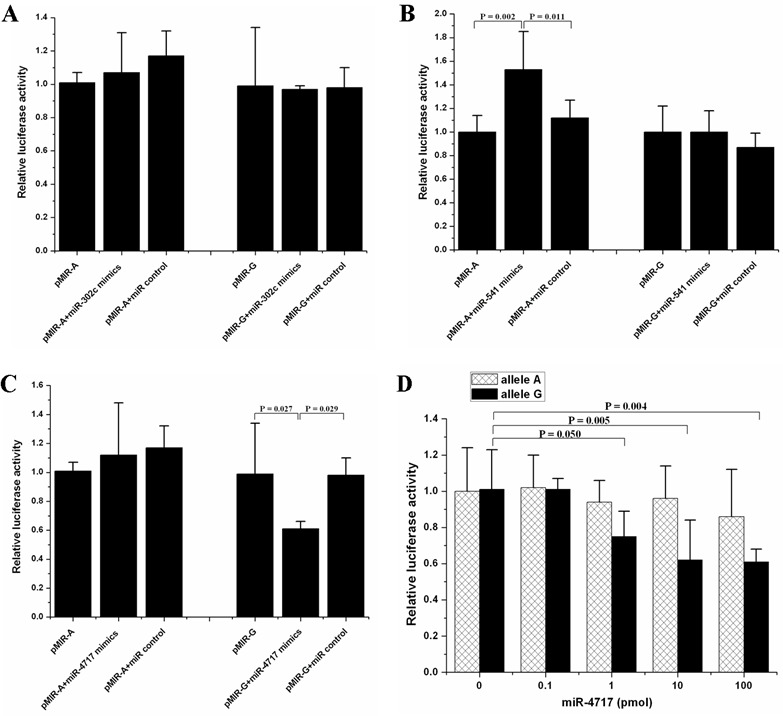
Effects of the predicted miRNAs on the luciferase activity of pMIR-A or pMIR-G bearing segments from *PD1* 3′UTR of rs10204525 allele A and allele G, respectively (**A**) Effects of miR-302c. (**B**) Effects of miR-541. (**C**) Effects of miR-4717. (**D**) Dose-response relationship between miR-4717 mimics concentration and luciferase activity of pMIR-G. Statistical analysis was performed by using one-way univariate analysis of variance (ANOVA) and the least significant difference (LSD) test.

Cotransfection of pMIR-A or pMIR-G with miRNA mimics of miR-302c, miR-541 and miR-4717 showed that miR-302c had no effect on the luciferase activity (Figure 2A). Cotransfection of miR-541 with pMIR-A significantly increased the luciferase activity (p = 0.002, compared with pMIR-A; p = 0.011, compared with pMIR-A + miR control, Figure 2B). Cotransfection of miR-4717 with pMIR-G significantly decreased the luciferase activity (p = 0.027, compared with pMIR-G; p = 0.029, compared with pMIR-G + miR control, Figure 2C). In contrast, the expression of the luciferase containing pMIR-A remains unchanged in the presence or absence of miR-4717 (Figure 2C).

miR-302c was not further investigated due to its null effect on the luciferase activity of both pMIR-A or pMIR-G transfected cells. miR-541 was also not further investigated because it had no significant effect on pMIR-G but pMIR-A transfected cells although it was predicted to putatively bind the 3′UTR of *PD1* mRNA with rs10204525 G allele. However, miR-4717 was further investigated owing to its predicted binding of the 3′UTR of *PD1* mRNA with rs10204525 G allele and its significant effect on pMIR-G luciferase activity as well as its null effect on the luciferase activity of pMIR-A transfected cells, indicating that miR-4717 may regulate PD-1 expression through differential interaction with 3′UTR of *PD1* mRNA with rs10204525 G allele or A allele.

The miR-4717 cotransfection at concentrations of 0, 0.1, 1.0, 10.0, and 100.0 pmol showed that the levels of pMIR-G luciferase activity gradually decreased with the increase of miR-4717 concentrations (p = 0.050, 1 pmol *vs.* 0 pmol; p = 0.005, 10 pmol *vs.* 0 pmol; p = 0.004, 100 pmol *vs.* 0 pmol, Figure 2D). These concentrations of miR-4717 showed no significant effect on the levels of pMIR-A luciferase activity (Figure 2D).

### Effect of miR-4717 on PD-1 mRNA expression in lymphocytes

**Figure 3:**
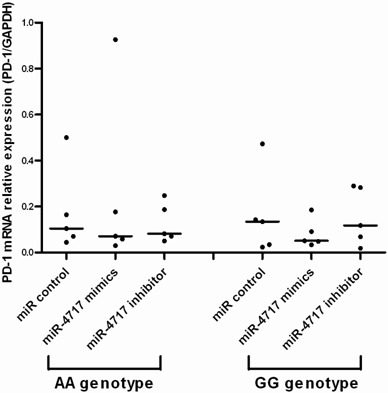
Effects of miR-4717 mimics or miR-4717 inhibitor on PD-1 mRNA expression in lymphocytes from chronic HBV patients with different rs10204525 genotypes assessed by real-time quantitative PCR Comparisons were performed by using non-parametric tests (Kruskal-Wallis H test and Mann-Whitney U test).

Assessed by real-time RT-PCR, transfection of miR-4717 mimics or miR-4717 inhibitors showed no significant effect on PD-1 mRNA levels in lymphocytes from chronic HBV patients with both *PD1* rs10204525 genotypes AA (n = 5) and GG (n = 5) (Figure 3).

### Effect of miR-4717 on PD-1 protein expression on lymphocytes

**Figure 4:**
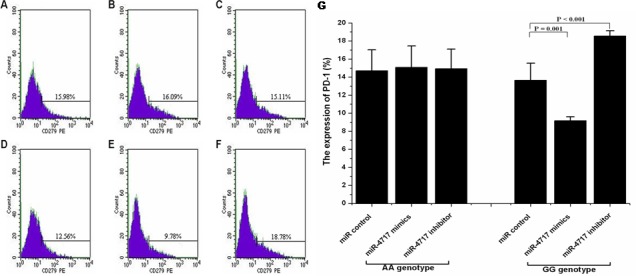
Effects of miR-4717 mimics or miR-4717 inhibitor on PD-1 protein expression on lymphocytes from chronic HBV patients with different rs10204525 genotypes assessed by flow cytometry (**A**) Genotype AA + miR control. (**B**) Genotype AA + miR-4717 mimics. (**C**) Genotype AA + miR-4717 inhibitor. (**D**) Genotype GG + miR control. (**E**) Genotype GG + miR-4717 mimics. (**F**) Genotype GG + miR-4717 inhibitor. (**G**) Comparison of PD-1 on lymphocytes from genotypes AA and GG by miR-4717 mimics and miR-4717 inhibitor. Statistical analysis was carried out by using one-way univariate analysis of variance (ANOVA) and Post Hoc test.

Compared with miR control (Figure 4D), transfection of lymphocytes from chronic HBV patients with *PD1* rs10204525 genotype GG (n = 5) with miR-4717 mimics significantly decreased PD-1 protein expression (p = 0.001, Figure 4E, Figure 4G), while transfection of the cells with miR-4717 inhibitor significantly increased PD-1 protein expression (p < 0.001, Figure 4F, Figure 4G) assessed by flow cytometry. Transfection of lymphocytes from chronic HBV patients with *PD1* rs10204525 genotype AA (n = 5) with miR-4717 mimics or miR-4717 inhibitor showed no such effects (Figure 4B, Figure 4C) compared with miR control (Figure 4A, Figure 4G).

### Effect of miR-4717 on TNF-α and IFN-γ production by lymphocytes

**Figure 5:**
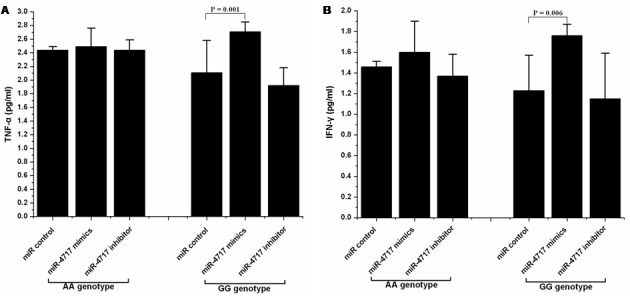
Effect of miR-4717 mimics or miR-4717 inhibitor on the production of tumor necrosis factor (TNF)-α and interferon (IFN)-γ by lymphocytes from chronic HBV patients with rs10204525 genotypes AA and GG (**A**) TNF-α. (**B**) IFN-γ. The values were log transformed to obtain normal distribution and then analyzed by using ANOVA and Post Hoc test.

Transfection of lymphocytes from chronic HBV patients with *PD1* rs10204525 genotype GG (n = 5) with miR-4717 mimics significantly increased TNF-α and IFN-γ levels (p = 0.001 and p = 0.006, respectively, compared with miR control, Figure 5A, Figure 5B). Transfection of lymphocytes from chronic HBV patients with *PD1* rs10204525 genotype GG genotype with miR-4717 inhibitor decreased, although not significantly, TNF-α and IFN-γ levels (p = 0.251 and p = 0.626, respectively, compared with miR control, Figure 5A, Figure 5B). Transfection of lymphocytes from chronic HBV patients with *PD1* rs10204525 genotype AA (n = 5) with miR-4717 mimics or miR-4717 inhibitor showed no similar findings (Figure 5A, Figure 5B).

### miR-4717 expression in peripheral lymphocytes of patients with chronic HBV infection

**Figure 6:**
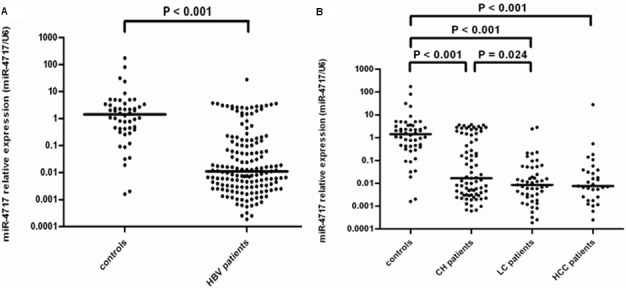
miR-4717 levels in patients with chronic HBV infection (**A**) Comparison of miR-4717 levels in patients with chronic HBV infection and health controls. (**B**) miR-4717 levels in HBV infected patients with different clinical diseases. Comparisons were performed by using non-parametric tests (Kruskal-Wallis H test and Mann-Whitney U test). HBV, hepatitis B virus; CH, chronic hepatitis; LC, liver cirrhosis; HCC, hepatocellular carcinoma.

The miR-4717 levels in lymphocytes from patients with chronic HBV infection (n = 157) were significantly decreased compared with those in controls (n = 50) (Mann–Whitney U test, p < 0.001, Figure 6A).

The miR-4717 levels between controls (n = 50) and patients with chronic hepatitis (n = 77), liver cirrhosis (n = 49) and HCC (n = 31) were significantly different (Kruskal-Wallis H test, p < 0.001). The levels in chronic hepatitis, cirrhosis and HCC were all significantly decreased compared with those in controls (Mann–Whitney Utest, all p < 0.001, Figure 6B). The miR-4717 levels in cirrhosis were significantly lower than those in chronic hepatitis (p = 0.024, Figure 6B).

### Association of miR-4717 with HBeAg status, HBV DNA and ALT levels

**Figure 7:**
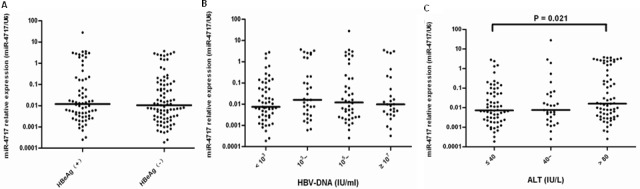
miR-4717 levels in HBV patients according to HBeAg status, HBV DNA and ALT levels (**A**) miR-4717 levels between HBeAg + and HBeAg – patients. (**B**) miR-4717 levels according to HBV-DNA loads. (**C**) miR-4717 levels according to between ALT concentrations. Comparisons were performed by using non-parametric tests (Kruskal-Wallis H test and Mann-Whitney U test).

In Mann-Whitney U test, the miR-4717 levels between HBeAg + (n = 68) and HBeAg – (n = 89) patients showed no significant difference (Figure 7A). By Kruskal-Wallis H test, the miR-4717 levels in HBV-DNA <10^3^ (n = 55), 10^3^∼ (n = 33), 10^5^∼ (n = 42) and ≥10^7^ (n = 27) also showed no significant difference (Figure 7B). By Kruskal-Wallis H test, the miR-4717 levels between ALT ≤ 40 IU/L (n = 63), 40∼ IU/L (n = 29) and > 80 IU/L (n = 65) were not significantly different (p = 0.054, Figure 7C). Further Mann–Whitney U test showed that miR-4717 levels in ALT ≤ 40 IU/L were significantly lower than in ALT > 80 IU/L (p = 0.021, Figure 7C).

### Association of miR-4717 with PD1 rs10204525 genotypes

**Figure 8:**
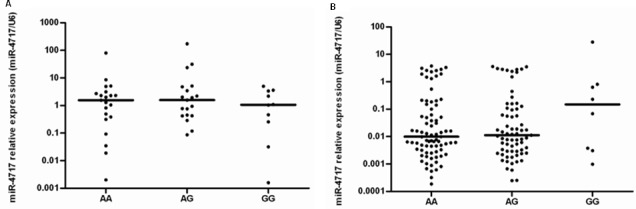
miR-4717 levels of peripheral lymphocytes in the study subjects with different *PD1* rs10204525 genotypes (**A**) miR-4717 levels in healthy controls. (**B**) miR-4717 levels in patients with chronic HBV infection. Comparisons were performed by using non-parametric tests (Kruskal-Wallis H test and Mann-Whitney U test).

In healthy controls, Kruskal-Wallis H test showed that miR-4717 levels between *PD1* rs10204525 genotypes AA (n = 21), AG (n = 19) and GG (n = 10) had no significant difference (p = 0.777, Figure 8A). Further Mann-Whitney U test between groups also showed no significant differences (Figure 8A).

In HBV patients, Kruskal-Wallis H test showed that miR-4717 levels between *PD1* rs10204525 genotypes AA (n = 80), AG (n = 69) and GG (n = 8) had no significant difference (p = 0.573, Figure 8B). Further Mann-Whitney U test between groups also showed no significant differences (Figure 8B).

## DISCUSSION

In this study, we predicted three miRNAs, miR-302c, miR-541 and miR-4717, which may putatively target the 3′ UTR of *PD1* mRNA with rs10204525 allele G in *PD1* gene. Of these predicted miRNAs, miR-4717 was shown to influence the luciferase activity containing *PD1* rs10204525 allele G in a dose-dependent manner. Transfection of lymphocytes from individuals with rs10204525 genotype GG with miR-4717 mimics significantly suppressed PD-1 protein expression on lymphocytes and upregulated the production of TNF-α and IFN-γ by lymphocytes. In contrast, these effects by miR-4717 were abolished in the luciferase activity containing *PD1* rs10204525 allele A and in lymphocytes from individuals with rs10204525 genotype AA. It is indicated that miRNA-4717 differentially interacts with its polymorphic target, rs10204525, in the *PD1* 3′ UTR. In patients with chronic HBV infection, miR-4717 levels in peripheral lymphocytes were significantly decreased in comparison with healthy controls. Patients with HBV-related cirrhosis had significantly lower miR-4717 levels than patients with chronic hepatitis B. It is indicated that miR-4717 is involved in the pathogenesis of chronic HBV infection.

miRNAs may regulate gene expression through sequence-specific interaction with the 3′ UTR of targeted mRNA at the post-transcriptional or translational level [32, 34]. In our study, transfection of lymphocytes from individuals with rs10204525 genotype GG with miR-4717 mimics had no significant influence on PD-1 mRNA expression, but this transfection significantly suppressed PD-1 protein expression on lymphocytes, suggesting that miR-4717 may regulate *PD1* gene expression primarily at the translational level.

Polymorphic miRNA target sites were shown to determine differences in gene expression [35]. Interestingly, the rs10204525 genotype GG was previously revealed to be associated with low susceptibility and disease progression [28] and low expression of PD-1 in chronic HBV infection [30], while the rs10204525 genotype AA was previously shown to be associated with higher susceptibility and disease progression [28] and higher expression of PD-1 [30]. The *PD1* rs10204525 was demonstrated to be a polymorphic miRNA target site in this study. Therefore, it is indicated that rs10204525 may differentially influence PD-1 expression, conferring an inhibitory or stimulatory effect on PD-1 function and thus reducing or enhancing its negative impact on T-cell activation and function, exhibiting a protective or predisposing role in chronic HBV infection [28].

In the present study, we have identified miR-4717 as regulator of *PD1* via binding to its target site in the 3′ UTR of *PD1* mRNA. To our knowledge, this is the first miRNA regulating *PD1* through interaction with SNP in the 3′ UTR of *PD1* gene reported thus far. miR-4717 suppressed PD-1 expression in a dose-dependent manner in luciferase activity assay and also suppressed PD-1 expression on lymphocytes from individuals with rs10204525 genotype GG. The suppression of PD-1 expression on lymphocytes was associated with increased production of TNF-α and IFN-γ. It is interesting to note that rs10204525 genotype GG in patients with chronic HBV infection had been previously shown to be associated with higher TNF-α and IFN-γ secretion [29]. It is indicated that the protective effect of rs10204525 genotype GG in chronic HBV infection may also, at least in part, relate to the inhibitory effect of miR-4717 on PD-1 expression and the subsequent increased production of TNF-α and IFN-γ through its interaction with mRNA of this specific polymorphic genotype. Numerous studies have shown that PD-1 negatively regulates the antiviral immune response and that cytokines, especially TNF-α and IFN-γ, play an important role in controlling HBV replication [2, 17, 23, 36, 37]. Of note, the effect of miR-4717 on PD-1 expression was abolished in rs10204525 genotype AA. Previous studies showed that rs10204525 genotype AA in patients with chronic HBV infection was associated with the disease progression and lower TNF-α and IFN-γ secretion [28, 29]. It is, therefore, suggested that the intrinsic non-response to miR-4717 may, at least partially, contribute to the disease course of HBV-infected individuals with rs10204525 genotype AA.

This study showed that the miR-4717 levels in patients with chronic HBV infection, especially in those with HBV-related cirrhosis, were significantly lower. These results are in line with the miR-4717 effect on PD-1 expression and cytokine production in this study, suggesting that miR-4717 plays a part in the pathophysiology of HBV infection and insufficient miR-4717 is associated with chronic HBV infection and possibly the development of fibrosis.

This study did not find any association between the rs10204525 genotypes and miR-4717 levels in both healthy control population and patients with chronic HBV infection, suggesting the possible differential expression of miR-4717. On the one hand, miRNAs may allele-specifically affect protein expression through sequence-specific impact. On the other hand, SNPs may also affect miRNA function, contribute to allele-specific protein expression [38]. Consequently, the influences of SNP rs10204525 and miR-4717 on chronic HBV infection and disease progression may be resulted from an imbalanced interaction between this SNP and miR-4717. Notably, previous studies have highlighted that both genetic variants and a miRNA expression patterns may be responsible for gene expression differences among distinct populations [38] and that target SNPs may contribute to disease susceptibility not *per se* [39, 40] but in coordination with miRNA expression patterns [38].

PD-1 plays vital roles in multiple biological and immunological processes involved in many diseases including viral infection and cancers. Breaking immune tolerance by therapeutic PD-1 blockade has been demonstrated to represent a therapeutic breakthrough and show broadly efficacious and durable cancer immunotherapies [41-44] as well as therapeutic potential in chronic viral infection [45]. To the best of our knowledge, there has been no miRNAs which are reported to affect the function of PD-1 via interaction with polymorphically specific mRNA at the 3′ UTR of *PD1* gene. Therefore, the findings in this study have provided novel target and genetic information which may be employed for designing novel therapeutic modalities against HBV infection and HBV-related disease including HCC and may also provide a biomarker for directing treatment options based on the manipulation of *PD1* gene expression or miR-4717 expression or both if the findings will be confirmed in more studies.

In conclusion, this study has identified a miRNA, miR-4717, that may allele-specifically regulate PD-1 expression through differential interaction with its polymorphic target in the *PD1* 3′ untranslated region and consequently play a role in the disease susceptibility of chronic HBV infection, providing novel mechanistic insights for the role of PD-1 and miR-4717 in HBV-associated diseases. In addition, in view of the importance of PD-1 in various viral and tumorigenic diseases, the findings in this study may also provide clues for mechanism of pathogenesis and rationale of therapeutic consideration for these disorders. Further studies are warranted to confirm and extend these findings.

## MATERIALS AND METHODS

### Patients and control subjects

Patients with chronic HBV infection were enrolled from the First Affiliated Hospital, School of Medicine, Xi'an Jiaotong University. Diagnosis of all cases were in line with the jointly revised diagnostic criteria by Branch Associations of Hepatology and Infectious and Parasitic Diseases of Chinese Medical Association [46] and Professional Committee of Liver Cancer of Chinese Anti-Cancer Association [47]. Patients with other liver diseases (viral hepatitis A, hepatitis C, hepatitis E, drug-induced liver injury, steatohepatitis, alcoholic hepatitis, autoimmune hepatitis and Wilson's disease), metabolic and endocrine diseases including diabetes mellitus and hyperthyroidism and human immunodeficiency virus infection, patients with serious comorbidities not related to the HBV infection such as cardiovascular, respiratory system and renal dysfunction and patients under 18 years of age were all excluded from the study. The control subjects were healthy individuals of Chinese Han population without kinship. Ethylenediaminetetraacetic acid (EDTA) anticoagulated whole blood was drawn early in the morning from the study subjects after overnight fasting. The total RNA and genomic DNA were immediately extracted, aliquoted and stored at −70 °C until use. The study was conducted in accordance with the Declaration of Helsinki, and the study protocol was reviewed by the ethics committee of the hospital. All subjects were voluntary to participate the study and provided informed consent.

### Prediction of miRNA by Bioinformatics

NCBI database (http://www.ncbi.nlm.nih.gov/snp/) was used to find genetic information of *PD1* gene including sequence around rs10204525 (+8669 A/G) site. The miRBase (http://www.mirbase.org/), Targetscan (http://www.targetscan.org/) and PicTar (http://pictar.mdc-berlin.de/) databases were used to predict miRNAs that may sequence-specifically target the 3′ UTR of *PD1* mRNA with different rs10204525 genotypes.

### Genomic DNA extraction and genotyping of PD1 rs10204525 polymorphism

Genomic DNA was extracted using TIANamp Genomic DNA Kit [Tiangen Biotech (Beijing) Co., Ltd., Beijing, China] according to the manufacturer's instructions. Genotyping of *PD1* rs10204525 (+8669 A/G) was performed by bidirectional polymerase chain reaction (PCR) amplification of specific alleles (Bi-PASA) using two outer and two inner allele-specific primers as described previously [28, 48].

### Construction of the vectors

Primers were designed with software oligo6.0 and primer5.0 according to sequence from Genbank (EF064716) and synthesized by Shanghai Sangon Biological Engineering Technology And Service Co., Ltd.(Shanghai, China). The sequences were upstream primer: 5′- GCGAAGCTTACCTGGGTGTTGGGAGGGCA −3′ and downstream primer: 5′- GCGACTAGTGGAGTGGATAGGCCACGGCG −3′. A Hind III and a Spe I restriction site (underlined) were introduced to the upstream primer and the downstream primer, respectively.

The partial sequences of 3′ UTR of *PD1* gene, which contain putative miRNA-binding sites, were amplified from genomic DNA by PCR using Taq Polymerase and Taq PCR Mix (Xi'an Runde Biotechnoly Co., Ltd., Xi'an, China). The PCR products were recruited by kit from TiangenBiotech (Beijing) Co., Ltd. (Beijing, China) and were then inserted into vector pMIR-REPORT^TM^ Luciferase (Ambion) after digestion with Hind III and Spe I [TAKARA Biotechnology (Dalian) Co., Ltd. Dalian, China]. The constructs were confirmed by double enzyme digestion with Hind III and Spe I (TAKARA Biotechnology (Dalian) Co., Ltd. Dalian, China) and sequencing. According to genotypes of *PD1* rs10204525 polymorphism, constructed vectors were named pMIR-A (containing allele A sequence) and pMIR-G (containing allele G sequence), respectively.

### miRNA mimics and inhibitor

miRNAs mimics and inhibitor were synthesized by Biomics Biotech (Nantong, China) and the sequences are shown in Supplementary Table 1.

### Dual luciferase reporter activity assay

HepG2 cells were cultured in RPMI 1640 medium (Hycolon) with 10% fetal calf serum and 1% penicillin-streptomycin and then in RPMI 1640 medium with 10% fetal calf serum without penicillin-streptomycin in an atmosphere of 5%CO_2_ at 37°C. Cells at logarithmic phase were seeded in 24-well plates at 1×10^5^ cells/well in an atmosphere of 5%CO_2_ at 37°C. The 70-80% confluent cells were cotransfected with 200 ng of the pMIR-A, pMIR-G, or 20 ng Renilla luciferase control plasmid (pRL-TK) (Promega, Madison, WI) and 10 pmol miRNA mimics of miR-302c, miR-541, miR-4717 or control miRNA (all diluted in 50 μl serum-free Opti-MEM medium) using Lipofectamine 2000 (Invitrogen, Carlsbad, CA) according to the instructions of the manufacturer.

Firefly luciferase activity was determined using the Dual Luciferase Assay system (Promega, Madison, WI) after cells were lysed with passive lysis buffer. The firefly and renilla luciferase activities were measured by fluorescence photometer (PerkinElmer). Relative luciferase activity was calculated by normalizing the firefly luciferase activity (pMIR-REPORT) to the internal control renilla activity (pRL-TK). The experiments were performed in quadruple.

### Isolation of peripheral blood lymphocytes and transfection

Lymphocyte Separation Medium (Human) (Applygen Technologies Inc. Beijing, China) was used to isolate peripheral blood mononuclear cells from EDTA anticoagulated fresh blood according to the manufacturer's instructions. Monocytes mixed with lymphocytes were removed by adhering glass of monocytes. The cell viability was evaluated by trypan blue staining.

Lymphocytes were adjusted to a concentration of 1×10^6^ cell/ml and cultured in RPMI 1640 medium for 72 hours. The cells were seeded in 24-well plates at 1 ×10^5^ cells/well and cultured under 5%CO_2_ at 37°C. The 30-50% confluent cells were transfected with 20 pmol of miR-4717 mimics, miR-4717 inhibitors or control miRNA using Lipofactamin 2000 (Invitrogen) according to the instructions. The experiments were performed in quadruple.

### RNA isolation

Total RNA from cells was isolated using the TRIzol reagent (Invitrogen, Carlsbad, CA) according to manufacturer's instruction after treatment with red blood cell lysis buffer [Tiangen Biotech (Beijing) Co., Ltd., Beijing, China]. After determination of the quality and concentration by measuring the optical density value at 260 nm and 280 nm, the RNA was stored at −70 °C until use.

### Determination of PD-1 mRNA levels

PD-1 mRNA levels were determined by real-time RT-PCR with primers as described elsewhere [30].

### Determination of miRNAs

The miRNA stem-loop primers were designed using reverse transcription PCR primer method [49]. The sequences of the primers designed for miR-4717 and U6 RNA are shown in Supplementary Table 2. The looped primers were used to detect miRNAs by real-time PCR. Total RNA was converted to cDNA by priming with a mixture of looped primers using RevertAid™ First Strand cDNA Synthesis Kit (Fermentas) according to the instructions of the manufacturer. Real-time PCR was performed using Maxima® SYBR Green/ROX qPCR Master Mix (Fermentas) based reaction containing corresponding primers in Real time PCR Detection System (IQ5, Bio-Rad). The U6 small nuclear RNA was included as internal reference. The relative expression levels of miRNA were calculated by 2^−ΔΔCT^ method [50]. All of the reactions were performed in triplicate.

### PD-1 expression on lymphocytes by flow cytometry

Lymphocytes were centrifuged at 100×g for 5 min and then washed with PBS. The cell viability was evaluated by trypan blue staining. The concentration of cells was adjusted to 1×10^6^ cell/ml. One ml of cell suspensions was used for interaction with Anti-Human CD279 (PD-1) PE monoclonal antibody (eBioscience, San Diego, CA) at 4 °C for 30 min. Cells were resuspended in 500 μl PBS for flow cytometry (FACSCalibur Flow Cytometer, Becton Dickinson Immuno-cytometry System, San Jose, CA, USA) after washing with PBS. Mouse IgG1 K Isotype Control PE antibody (eBioscience, San Diego, CA) was used as a negative control.

### Determination of TNF-α and IFN-γ

Levels of TNF-α and IFN-γ were determined using commercially available Human TNF-α and IFN-γ ELISA kits (R&D Systems, Inc., Minneapolis, MN, USA), respectively.

### Statistical analysis

SPSS16.0 software (SPSS Inc. Chicago) was used for statistical analysis. Relative luciferase activity was compared using one-way univariate analysis of variance (ANOVA). Comparisons between groups were performed using the least significant difference (LSD) test. Data of flow cytometry were analyzed using one-way ANOVA. Comparisons between groups were carried out using Post Hoc test. TNF-α and IFN-γ values were log transformed to the normal distribution and analyzed using ANOVA. Comparisons between groups were carried out using Post Hoc test. The t test or χ2 test was used to compare the data between HBV infection and control groups. The comparisons of expression levels of mRNA or miRNA between groups were performed by non-parametric tests (Kruskal-Wallis H test and Mann-Whitney U test). A *P* value < 0.05 was considered statistically significant.

## SUPPLEMENTARY FIGURES AND TABLES


