# Supplementary material for: microRNA-4717 differentially interacts with its polymorphic target in the PD1 3′ untranslated region: A mechanism for regulating PD-1 expression and function in HBV-associated liver diseases
Source: Oncotarget. 2015 Mar 26;6(22):18933–44. doi: 10.18632/oncotarget.3662 (PMC4662465; doi:10.18632/oncotarget.3662)
Supplement: Supplementary file 1 [file oncotarget-06-18933-s001.pdf]

microRNA-4717 differentially interacts with its polymorphic target in the *PD1* 3′ untranslated region: A mechanism for regulating PD-1 expression and function in HBV-associated liver diseases

Supplementary Material

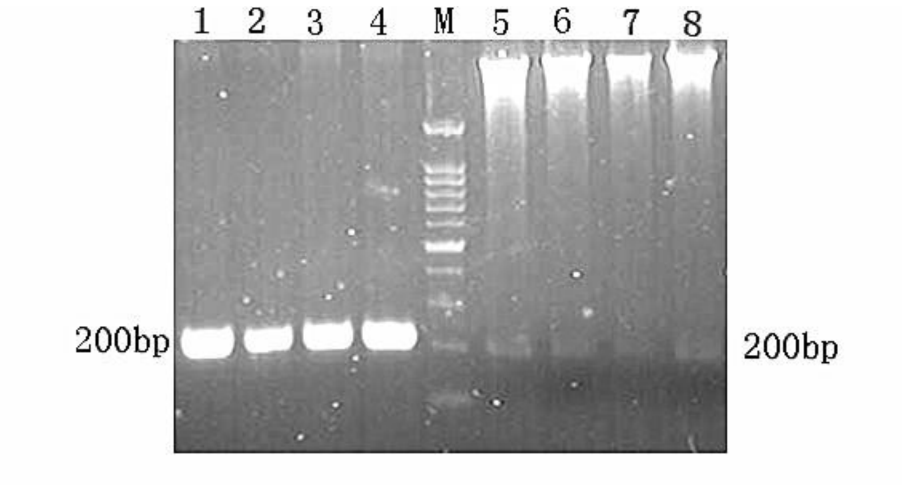

Supplementary Figure 1: pMIR-REPORT-PD1 identified by PCR and restriction enzyme digestion. M: 100bp DNA Marker; 1-4: PD1 fragment; 5-8: pMIR-REPORT-PD1 vector digested with Hind Ⅲ and Spe I.

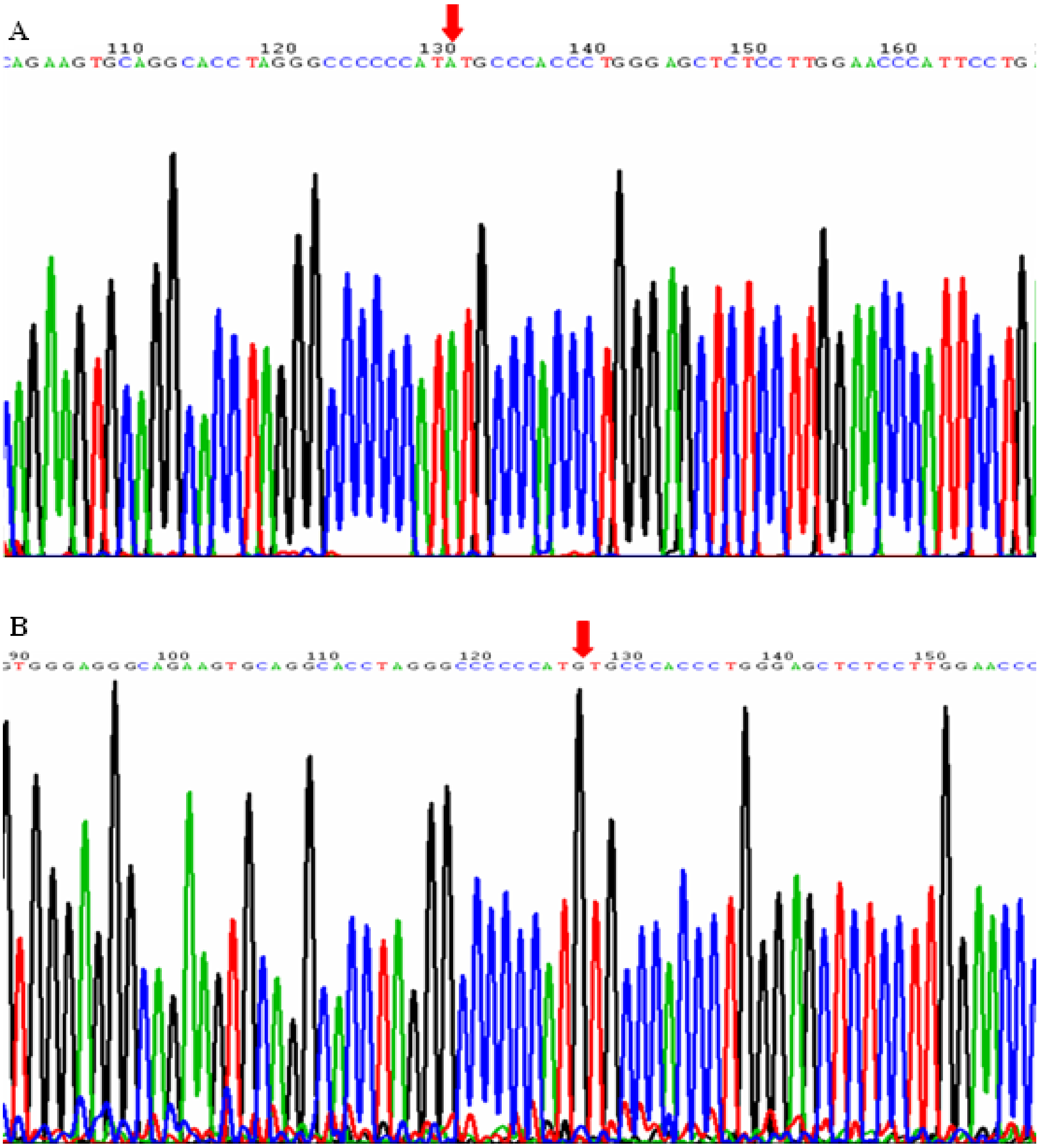

Supplementary Figure 2: pMIR-A and pMIR-G partial results of sequencing. **(A)** pMIR-A. **(B)** pMIR-G.

Supplementary Table 1: The sequences of the miRNA mimics and inhibitor

miRNA		Sequence (5’-3’)
miR-302c mimics	Sense strand	UUUAACAUGGGGGUACCUGCUG
	Antisense strand	CAGCAGGUACCCCCAUGUUAAA
miR-541 mimics	Sense strand	UGGUGGGCACAGAAUCUGGACU
	Antisense strand	AGUCCAGAUUCUGUGCCCACCA
miR-4717 mimics	Sense strand	ACACAUGGGUGGCUGUGGCCU
	Antisense strand	AGGCCACAGCCACCCAUGUGU
miRNA control	Sense strand	UUCUCCGAACGUGUCACGUTT
	Antisense strand	ACGUGACACGUUCGGAGAATT
miR-4717 inhibitor		AGGCCACAGCCACCCAUGUGU

Supplementary Table 2: The sequences of primers for miR-4717 and U6 RNA

	Primer sequences	Product (bp)
hsa-miR-4717	Reverse primer：<br>5’-GTCGTATCCAGTGCAGGGTCCGAGGTATTCGCACTGGA<br>TACGACAGGCCA-3’<br>Upstream primer 5’-CGCCACACATGGGTGGCTG-3’<br>Downstream primer 5’-GTGCAGGGTCCGAGGT-3’	60bp
U6 RNA	Upstream primer：5’-CTCGCTTCGGCAGCACA-3’<br>Downstream primer：5’-AACGCTTCACGAATTTGCGT-3’	94bp
